# Bioactive Compounds of Shrimp Shell Waste from *Palaemon serratus* and *Palaemon varians* from Portuguese Coast

**DOI:** 10.3390/antiox12020435

**Published:** 2023-02-09

**Authors:** Maria Luz Maia, Clara Grosso, M. Fátima Barroso, Aurora Silva, Cristina Delerue-Matos, Valentina Fernandes Domingues

**Affiliations:** 1 REQUIMTE/LAQV, ISEP, Polytechnic of Porto, Dr. António Bernardino de Almeida, 4249-015 Porto, Portugal; 2 Nutrition and Bromatology Group, Department of Analytical and Food Chemistry, Faculty of Food Science and Technology, Ourense Campus, University of Vigo, E32004 Ourense, Spain

**Keywords:** shrimp characterization, waste valorization, antioxidant activity, neuroprotective activity, antimicrobial activity

## Abstract

The production and consumption of shrimp species create massive amounts of shrimp bio-waste. In this study, shrimp shell waste from *Palaemon serratus* and *Palaemon varians* from the Portuguese coast was characterized. Regarding the antioxidant capacity, the obtained values were between 4.7 and 10.4 mg gallic acid equivalents (GAE)/g dry weight (dw) for Total phenolic content (TPC); 3 and 7 mg ascorbic acid equivalents (AAE)/g dw for Ferric reducing antioxidant power assay (FRAP); 0.4 and 1.2 mg Trolox equivalent (TE)/g dw for 2,2-diphenyl-1-picryl-hydrazyl-hydrate free radical scavenging (DPPH^•^); 4 and 11 mg TE/g dw for 2,2′-azino-bis (3-ethylbenzothiazoline-6-sulfonic acid) radical scavenging activity (ABTS^•+^); and 72 and 130 mg TE/g dw for Oxygen radical absorbance capacity (ORAC). For the antimicrobial activity, shrimp shell waste from *P. varians* formed inhibition zones between 14 and 23 mm. Total carotenoid content values were in the range of 28 and 134 μg/g dw, and according to their HPLC-PAD profile, β-carotene and astaxanthin contents were between 0.3 and 7.6 μg/g dw and 1.1 and 26.1 μg/g dw, respectively. These studies are critical to recognizing the potential added value of shrimp shell waste as possible colorants and preservatives with antioxidant protection capacity to be used in the food industry.

## 1. Introduction

Shrimps are crustaceans that are highly appreciated all over the world, suggested as high-quality food, and considered part of a balanced diet. The shrimp market has been growing over the years, which makes shrimps a valuable source of nutrients for the human population [1]. Shrimps are a good source of protein, vitamins, astaxanthin, and minerals such as phosphorus, calcium, selenium, copper, zinc, and iodine [2]. The Food and Agriculture Organization (FAO) establishes that shrimp consumption in several countries has been increasing globally. The FAO database reports that 12,611,809 tons of shrimp were consumed in 2020 in the world, and this value in Portugal was 28,835 tons. These data highlight the importance of shrimp in the global economy and also the amount of shell waste produced. Furthermore, Portugal's rate of consumption is higher than the worldwide average, with the quantity consumed per capita in 2020 being 2.83 kg in Portugal compared to the global average of 1.63 kg [3,4]. Regarding the capture of shrimp species in Portugal, in 2020 it reached 87 tons. This amount provided revenues of EUR 2,565,000 [5]. 

Depending on market demand, shrimps can be exported with or without the shell. All this consumption and production generates massive amounts of shrimp bio-waste. Usually, this waste comprises approximately 50% of the weight of raw shrimp [6]. The increase in the disposal of shrimp waste in the surrounding environment is a critical contribution to environmental pollution and health risks. Consequently, the processing and application of shrimp shell waste are crucial to reducing environmental pollution [6,7]. Pigments with high antioxidant capacities from shrimp shell wastes can be a good added value for new bioproducts, for instance, in the food industry, since shrimp shells are a good source of protein, minerals, carotenoids, such astaxanthin, a powerful antioxidant, and enzymes [7,8]. The shell composition of crustaceans, such as shrimp, can be slightly different depending on the species, season, and habitat; nevertheless, the main composition is calcium carbonate (20–50%), protein (20–40%), chitin (15–40%), and lipids (0–14%) with elevated contents of omega-3 fatty acids and pigments [9]. Several extractions have been performed to extract the pigments like carotenoids, and more specifically astaxanthin, from shrimp shell waste. Ethanol was considered suitable, effective, and safe to perform this extraction [10]. 

The current work aimed to explore the antioxidant, neuroprotective, and antimicrobial activities of shrimp shell waste. For that purpose, two shrimp species, *Palaemon serratus* and *Palaemon varians,* collected in different seasons and locations on the Portuguese coast, were studied. To the best of our knowledge, this is the first study assessing the influence of the seasons and sampling locations on the production of carotenoids in these two species from the Portuguese coast. 

## 2. Materials and Methods

### 2.1. Reagents and Materials

Folin-Ciocalteu, 2,2-diphenyl-1-picryl-hydrazyl-hydrate free radical scavenging (DPPH), 6-hydroxy-2,5,7,8-tetramethylchroman-2-carboxylic acid (Trolox) dimethyl sulfoxide (DMSO), lactic acid, ascorbic acid, 2′,2′-azobis (2-amidinopropane) dihydrochloride (AAPH), α-glucosidase, α-amylase, butyrylcholinesterase (BuChE), acetylcholinesterase (AChE), 4-nitrophenyl-α-D-glucopyranoside, acarbose, Tris-HCl, potassium phosphate monobasic, potassium phosphate dibasic trihydrate**,** bovine serum albumin (BSA), galantamine, acarbose, starch, 3,5-dinitrosalicylic acid, and astaxanthin standard (≥97% (high performance liquid chromatography, HPLC), from *Blakeslea trispora*) were purchased from Sigma Aldrich (Steinheim, Germany). Gallic acid was obtained from Fluka (Steinheim, Germany). β-carotene standard (≥95% (HPLC) was from Extrasynthese, (Genay, France). Sodium hydroxide was from Labkem (Barcelona, Spain). Sodium carbonate was from Panreac (Barcelona, Spain). Potassium sodium tartrate tetrahydrate was from Scharlab (Barcelona, Spain). The culture media Mueller–Hinton broth (MHB) and Mueller–Hinton II agar were acquired from Sigma-Aldrich (Madrid, Spain). Ethanol absolute and butyl methyl ether were from Carlo Erba (Val de Reuil, France), and methanol was from Riedel-de Haёn (Seelze, Germany). Ultrapure water resistivity of 18.2 MΩ cm, was from a Simplicity 185 water purification system (Millipore, Molsheim, France). All the solvents used were HPLC grade with the quality available (95–99%). All the spectrophotometric assays were performed in a Synergy HT W/TRF Multi Mode Microplate Reader with Gen5 2.0 software (BioTek Instruments, Winooski, VT, USA). 

### 2.2. Samples

Shrimp shell waste from two different shrimp species from the Portuguese coast was analyzed. *P. serratus* was gathered at Figueira da Foz and Vila do Conde by local fishermen, and *P. varians* was from wild and aquaculture origins gathered in the Sado estuary. These two species are well described in the literature [11]. The sampling locations were chosen to represent different locations between the north and the center of Portugal. Northern waters are colder, and the Sado estuary represents a site with brackish water against the salted water from the other two locations (Figueira da Foz and Vila do Conde). Sampling was carried out in autumn and spring between 2017 and 2019, according to the period allowed for the capture [12]. The shell was separated from the edible portion of the shrimp and analyzed.

### 2.3. Extraction Procedure

Shrimp shells were dehydrated at 41 °C (Excalibur, model 4926T, Dublin, Ireland) for 48 h and grounded to obtain reduced particles. Preliminary extractions were carried out where different solvents (Ethanol, 50% Ethanol and 70% Acetone), different solid: solvent ratios (5 g:50 mL and 5 g:100 mL), and different temperatures (room temperature and 40 °C) were tested. The best condition was 5 g of shrimp shell and 50 mL of absolute ethanol solution under agitation for 2 h at room temperature. In terms of Total Phenolic Content (TPC), this extraction was not statistically different from the other three conditions: with absolute ethanol using 100 mL (instead of 50 mL) at room temperature or applying 40 °C, and the extraction performed with acetone, 5 g/50 mL of solid-solvent ratio and 40 °C. Moreover, considering Ferric Reducing Antioxidant Power (FRAP) assays, extractions performed with absolute ethanol were statistically and significantly different from all the others performed. Altogether, considering three fundamental aspects related to the extraction processes, such as the environmental impact of using higher volumes of solvent, the costs associated with the use of temperature, and that ethanol is considered a GRAS solvent (Generally recognized as safe), the extraction condition using absolute ethanol, at room temperature, and with a solid–solvent ratio of 5 g/50 mL was selected. After that, the content was filtered with filter paper and then evaporated at 40 °C under a vacuum and protected from light in a rotary evaporator. 

Calibration standards for all the analyses were prepared daily, and all extracts were analyzed in triplicate. An r^2^ ≥ 0.99 was found for all calibration curves.

### 2.4. Chemical Characterization of the Extracts

#### 2.4.1. Total Phenolic Content

TPC determination was performed based on the Folin–Ciocalteu procedure [13]. Gallic acid (GA) was used as standard, and the absorbance was measured at 760 nm. TPC was expressed as mg of GA equivalents per g of dry weight (dw) of extract (mg GAE/g extract dw).

#### 2.4.2. Total Carotenoids Content

Total Carotenoids Content (TC) determination in the methanolic extracts was assessed by a colorimetric method and expressed in µg/g dw [14]. Contents were calculated using Equation (1), where A_480_ and A_750_ were the absorbances recorded at 480 and 750 nm, respectively:

Carotenoid (µg/mL) = 4 × (A_480_ − A_750_) (1)

#### 2.4.3. High Performance Liquid Chromatography (HPLC) with A Photodiode Array Detector (PDA)

The eluents used in the HPLC unit (Shimadzu, Kyoto, Japan) equipped with a photodiode array detector (PDA) model SPD-M20A were filtered through a 0.22 μm nylon membrane filter (Fioroni Filters, Ingré, France) using a vacuum pump (Dinko D-95, Barcelona, Spain) and degassed for 15 min in an ultrasonic bath (Sonorex Digital 10P, Bandelin DK 255P, Germany). Twenty μL of each shrimp shell extract were injected on an analytical HPLC with a low-pressure quaternary pump (model LC-20AT), a degasser (model DGU-20A5R), an auto-sampler (model SIL-20AT), and a column oven (model CTU-20AC). The gradient and column were previously described [15] and were used with some modifications. Briefly, compound separation was achieved using a C30 YMC Carotenoid S-5µm (25.0 × 0.46 cm; 5 μm particle size) column (Kyoto, Japan). The solvent system consisted of methanol (A) and MTBE (B) starting with 95% A, using a gradient to obtain 70% A at 30 min, 50% A at 60 min, and again 95% A at 65 min. The solvent flow rate was 0.9 mL/min. Chromatograms were recorded at 450 nm, and data were processed on LabSolutions software. Compounds were identified by comparing their UV–vis spectra and retention times with standards injected in the same conditions.

### 2.5. Biological Activity of the Extracts

#### 2.5.1. Antioxidant Capacity

Ferric Reducing Antioxidant Power

FRAP determination was performed according to the previously developed method by Paz et al. [13]. Ascorbic acid (AA) was used as the standard, and the absorbance was measured at 593 nm. FRAP results were expressed as mg of AA equivalents per g dw of extract (mg AAE/g dw). 

2,2-Diphenyl-1-Picryl-Hydrazyl-Hydrate Free Radical Scavenging

DPPH^•^ scavenging activity was performed as previously described [16]. Trolox solution was used as the standard, and the absorbance was measured at 517 nm. The results were expressed as mg of Trolox equivalents per g of dw of extract (mg TE/g dw). Inhibition (%) of DPPH activity was calculated after subtracting the respective blanks. The results were also expressed as the concentration needed to inhibit a biological process by 50% values (IC_50_).

2,2′-Azino-Bis (3-Ethylbenzothiazoline-6-Sulfonic Acid) Radical Scavenging Activity

2,2′-azino-bis (3-ethylbenzothiazoline-6-sulfonic acid) radical scavenging activity (ABTS^•+^) was determined according to Correia et al. [17]. A trolox solution was used as the standard and the absorbance was measured at 734 nm [17]. The results were expressed as mg of Trolox equivalents per g of dw of extract (mg TE/g dw). Inhibition (%) of ABTS^•+^ scavenging activity was calculated. The results were also expressed as IC_50_ values.

Oxygen Radical Absorbance Capacity

Oxygen radical absorbance capacity (ORAC) was performed as previously described [18]. Trolox solution was used as the standard. Briefly, disodium fluorescein (FL) was added to sample dilutions, and the resulting mixture was equilibrated for 30 min at 37 °C. Then, the reaction was initiated by the addition of AAPH, and the fluorescence was recorded. The results were expressed as mg of Trolox equivalents per g of extract (mg TE/g dw).

#### 2.5.2. Enzyme Inhibition

AChE and BuChE Inhibition Activities

AChE and BuChE inhibition activities of shrimp shell extracts were determined according to Ellman’s method [19] with slight modifications. Briefly, samples were dissolved in 50 mM Tris–HCl buffer (pH 8) with 10% MeOH. Then, 25 µL of the samples previously dissolved were added to the wells with 125 µL of 3 mM DTNB reagent, 50 µL of 50 mM Tris–HCl buffer (pH 8) with 0.1% of BSA, 25 µL of ATCI/BTCI, and 25 µL of 0.44 U/mL of AChE or 0.40 U/mL of BuChE. The initial slopes of the reaction were calculated at 405 nm and the Tris–HCl buffer with 10% of MeOH was used as a negative control. The values for inhibitory activity were calculated. Galantamine was used as the positive control (IC_50_ = 0.92 μg/mL against AChE and IC_50_ = 4.92 μg/mL against BuChE).

α-Glucosidase Inhibition Activity

α-Glucosidase inhibition activity of shrimp shell extracts was determined as previously described [20]. Samples were dissolved in potassium phosphate buffer 10 mM (pH 7), then 50 µL were added to the wells. Next, 130 µL of buffer was added, plus 100 µL of enzyme substrate (4-nitrophenyl-α-D-glucopyranoside 2.5 mM) and 20 µL of α-glucosidase. The microplate was incubated at 37 °C, and the reaction slopes were calculated recording the absorbance at 405 nm for 10 min. Potassium phosphate buffer 10 mM was used as the negative control and acarbose as a positive control (IC_50_ = 302 µg/mL), and the values for inhibitory activity were calculated. 

α-Amylase Inhibition Activity

α-Amylase inhibition activity of shrimp shell extracts was determined as previously described [21]. Samples were dissolved in 20 mM sodium phosphate buffer (pH 6.9 with 6 mM sodium chloride). Briefly, 100 μL of the sample plus 100 μL of 1% starch solution in buffer were incubated at 25 °C for 10 min. Next, 100 μL of α-amylase (8 mg/mL) was added and incubated again at 25 °C for 10 min. After adding 200 μL of dinitro alicyclic acid (DNS) color reagent, the reaction was stopped by incubating at 100 °C for 5 min. After the samples cooled down, 50 μL was removed from each tube and transferred to a microplate and 200 μL of water was added to each well. Absorbance was measured at 540 nm. Acarbose was used as the positive control. Sodium phosphate buffer 20 mM (pH 6.9 with 6 mM sodium chloride) was used as a negative control, and the values for inhibitory activity were calculated. 

#### 2.5.3. Antimicrobial Activity

The shrimp shell extracts were dissolved in DMSO (45 mg/mL), and the evaluation of antimicrobial activity was performed as described previously [22]. The antimicrobial properties of the shrimp shell extracts were tested against the following bacterial strains: Gram-positive *Escherichia coli* (NCTC 9001) that were supplied by Microbiologics, Minnesota USA, and *Pseudomonas aeruginosa* (ATCC 10145), *Salmonella enteritidis* (ATCC 13676), Gram-negative *Staphylococcus aureus* (ATCC 25923), and *Bacillus cereus* (ATCC 14579) were provided by Selectrol (Buckingham, UK). The determination of antimicrobial activity was achieved via Petri dish culture and measurement of the inhibition by the production of halos after the addition of the extracts [23]. Primarily, the bacteria were inoculated with MHB and were left to grow between 12 h and 24 h at 37 °C. Employing UV spectrometry at 600 nm, the number was set between 1 and 2 × 10^8^ colony forming units (CFUs). For that purpose, an absorbance between 0.09 and 0.110 of the McFarland scale was used. Briefly, 50 μL of the cultivated inoculum of the different bacteria was deposited and streaked. The Petri dish was divided into 4 quadrants, and 15 μL of 40% lactic acid was added as a positive control. In the remaining quadrants, 15 μL of the extract was added, and 15 μL of DMSO was added as a negative control in the center of the Petri dish and incubated for 24 h at 37 °C. The halos resulting from the inhibition were measured using a pachymeter in millimeters [24].

### 2.6. Statistical Analysis

All the experiments were repeated at least three times and presented as mean±standard deviation (SD). The statistical significance of the difference between the two groups was assessed by the nonparametric Mann–Whitney test, whereas the differences between more than two groups were assessed by Kruskal–Wallis one-way analysis of variance using Graph Pad Prism Software, version 6. The IC_50_ values were calculated also using Graph Pad Prism Software, version 6. The Pearson correlation coefficient was used to evaluate the correlation between different elements analysed. For that, SPSS Inc., version 22.0 was used (SPSS Inc., Chicago, IL, USA). Differences were considered statistically significant for *p* < 0.05.

## 3. Results

### 3.1. Chemical Characterization of the Extracts

Table 1 displays the TPC and TC values for the various locations and seasons; all data are reported in g dw of extract.

**Figure 1:**
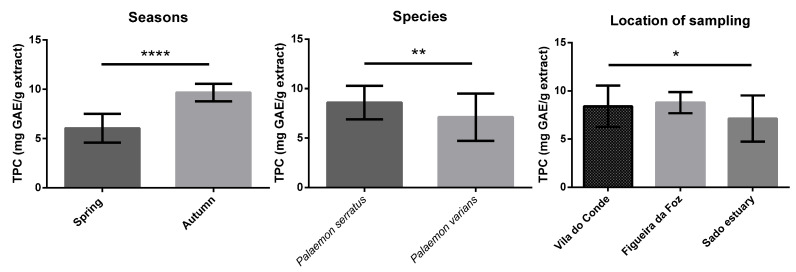
Differences in TPC content between seasons, species, and locations. Columns correspond to mean of the concentration ± SD. Significantly different, **** *p <* 0.0001; ** *p <* 0.01, * *p <* 0.05.

**Figure 2:**
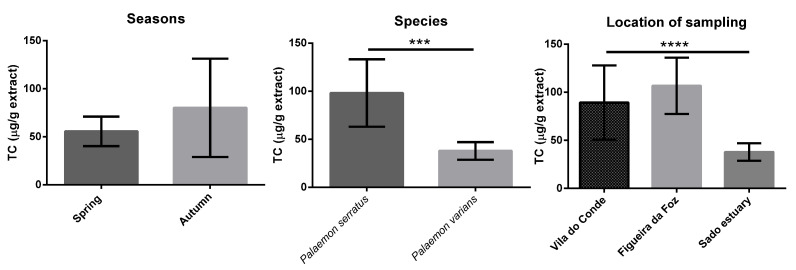
Differences in TC content between seasons, species, and locations. Columns correspond to mean of the concentration ± SD. Significantly different, **** *p <* 0.0001, *** *p <* 0.001.

**Figure 3:**
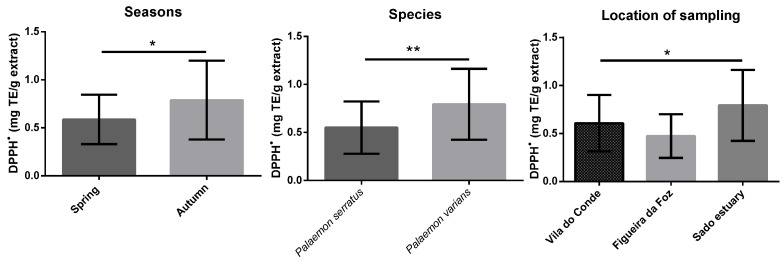
Differences of DPPH^•^ scavenging activity between seasons, species, and locations. Columns correspond to mean of the concentration ± SD. Significantly different, ** *p* < 0.01, * *p* < 0.05.

**Figure 4:**
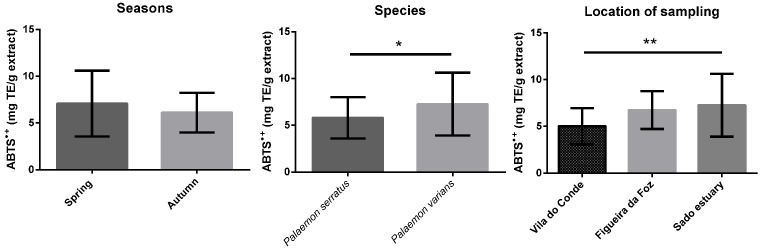
Differences of ABTS^•+^ scavenging activity between seasons, species, and locations. Columns correspond to mean of the concentration ± SD. Significantly different, ** *p* < 0.01, * *p* < 0.05.

Figure 1, Figure 2, Figure 3, Figure 4 and Figures S2–S5 display the comparison between seasons, species, and locations of sampling. Regarding seasons, all samples collected in spring were compared with those collected in autumn. For species comparison, all *P. serratus* samples were compared with those of *P. varians*, regardless of their location or season. Finally, all samples of *P. serratus* (spring + autumn) collected in Figueira da Foz, Vila do Conde and of *P. varians* (Spring + Autumn) collected in the Sado estuary were treated as independent groups.

For TPC content, the mean values ranged between 4.7 and 10.4 mg GAE/g dw. Regarding the influence of season, species, and location of sampling, as shown in Figure 1, the values were significantly higher in autumn (9.7 mg GAE/g dw, *p* < 0.0001), in *P. serratus* (8.6 mg GAE/g dw, *p* < 0.01), and collected from Figueira da Foz (8.8 mg GAE/g dw, *p* < 0.05), respectively. 

TC average values ranged between 28 and 134 μg/g dw (Table 1). Statistically significant differences were observed concerning the species and locations of sampling (Figure 2). The values were higher in *P. serratus* at 98 μg/g dw (*p* < 0.0001) and Figueira da Foz at 107 μg/g dw (*p* < 0.0001). 

Regarding the HPLC profile of the analyzed shrimp shell extract, β-carotene, and astaxanthin were identified and quantified, and an example of a chromatogram is presented in Figure S1. β-carotene and astaxanthin concentrations obtained in the shrimp extracts are presented in Figures S2 and S3. β-carotene mean values ranged between 0.3 and 7.6 μg/g dw. Regarding the different seasons, the values were significantly higher in spring at 4.1 μg/g dw (*p* < 0.001), and for different species and locations of sampling, no statistically significant differences were observed (Figure S2). Concerning astaxanthin, the mean values ranged between 1.1 and 26.1 μg/g dw. This time, no significative differences were observed for the different seasons (spring and autumn), whereas significative differences were observed when compared between different species and different locations of the sampling (Figure S3). Regarding shrimp species, the values were higher in *P. serratus* 17.3 μg/g dw (*p* < 0.0001), and, in the case of location of sampling, the values were higher in Vila do Conde 18.8 μg/g dw (*p* < 0.001). 

### 3.2. Biological Activity of the Extracts

The results regarding FRAP, DPPH^•^, ABTS^•+^, and ORAC measurements are presented in Table 2 for the different locations and seasons. All the results are presented by g dw of extract. 

Regarding FRAP, the mean values were between 3 and 7 mg AAE/g dw (Table 2). As presented in Figure S4, the values were significantly higher in autumn as well, with a value of 6.3 mg AAE/g dw (*p* < 0.0001). For species and location, no statistically significant differences were observed. 

Concerning DPPH^•^ scavenging activity, the mean values ranged between 0.4 and 1.2 mg TE/g dw, and for IC_50_ values ranged between 1.7 and 8.2 mg/mL (Table 2). Figure 3 shows that the values were significantly higher in autumn as well at 0.79 mg TE/g dw (*p* < 0.05), in *P. varians*, 0.79 mg TE/g dw (*p* < 0.01), and in Sado estuary, 0.79 mg TE/g dw (*p* < 0.05).

ABTS^•+^ scavenging activity mean values were between 4 and 11 mg TE/g dw, with IC_50_ values ranging from 0.3 to 0.6 mg/mL. In the case of ORAC mean values, these ranged between 72 and 130 mg TE/g dw (Table 2). As well as for DPPH^•^, Figure 4 and Figure S5 show that the values for ABTS^•+^ and ORAC were significantly higher in *P. varians,* 7.3 mg TE/g dw (*p* < 0.05) and 98.8 mg TE/g dw (*p* < 0.001), respectively, and in Sado estuary, 7.3 mg TE/g dw (*p* < 0.01), and 98.8 mg TE/g dw (*p* < 0.01), respectively. 

### 3.3. Enzyme Inhibition

Concerning the determination of the enzyme inhibition by the shrimp shell extracts, the IC_50_ values were not possible to calculate due to the weak inhibition power of the extracts. Concentrations of the shrimp shell extracts tested were between 600 and 150 μg/mL for AChE and BuChE, between 250 and 20 μg/mL for α-glucosidase, and between 380 and 20 μg/mL for α-amylase. AChE and BuChE inhibitions were lower than 15% for 600 μg/mL of the extract. Concerning α-glucosidase, the inhibition was lower than 20% for 250 μg/mL of the extract, and for α-amylase the inhibition was lower than 10% concerning the concentration of 380 μg/mL of the extract. 

### 3.4. Antimicrobial Activity

The results regarding the inhibition zones obtained for the shrimp shell extracts against several bacteria are presented in Table S1. The classification proposed by the Clinical and Laboratory Standards Institute was used for this assay, where the diameter of the inhibition growth measured allows the classification of bacteria as resistant (R, ≤ 14 mm), intermediate (I, 15–19 mm), or susceptible (S, ≥ 20 mm) [23]. 

Only the shrimp shell extracts from the Sado estuary (*P. varians*) formed inhibition zones. In the case of *P. aeruginosa*, both wild and aquaculture origin extracts were able to develop inhibition zones ranging from 14 to 20 mm (Table S1). *B. cereus* and *S. enteritidis* were also significative inhibited by *P. varians* shells extract (aquaculture origin). The inhibition zones in the case of *B. cereus* varied between 21 and 23.3 mm and for *S. enteritidis* ranged between 18 and 20 mm (Table S1). The results also demonstrate that the tested extracts do not show any activity against *E. coli* and *S. aureus*. 

### 3.5. Correlations between Elements Analyzed

The data described in Table S2 show a strong Pearson correlation (0.5 < |r| < 1) and *p* ˂ 0.05 between the levels of the elements analyzed. Positive correlations were found between the values of TPC and FRAP and also between the values of TC and astaxanthin. However, negative correlations were found between the values of DPPH^•^ scavenging and astaxanthin and also between values of FRAP and β-carotene. 

## 4. Discussion

Regarding chemical characterization (TPC and TC), higher values were obtained for *P. serratus*, in autumn, and in Figueira da Foz, and astaxanthin values were also higher in *P. serratus*. Some of these results could be explained by the size of the shrimp because *P. serratus* was between 4 and 9 cm, *P. varians* was between 2 and 4 cm, and their metabolic rate could be different. *P. serratus*, in this case, seems to be producing more phenolic and carotenoid compounds (total and astaxanthin). Carotenoid retention was previously described to be dependent on the species analyzed and their reproduction cycle [25,26]. Seasonal variation of the TC could also be explained by changes in the quantity and quality of the algae available to the shrimp diet [25]. The results found in this work were slightly lower than the previously reported TPC value of 38 mg GAE/g dw [27]. The differences in the TPC values found between this study and previously reported ones might be attributed to variances in shrimp species or changes in the sampling site. The study of Shiekh et al. [27] used shrimp shells from a fish market in Jeddah, Saudi Arabia, and the solvent used in the extraction was methanol, whereas the current study used shrimps from the Portuguese coast and ethanol as the extraction solvent. So, as shown in the current study, different locations and species can have different impacts on TPC values. Regarding TC, a previous study reported TC mean values lower than the values determined in the present study [28], in this previous study, three shrimp species, *Farfantepenaeus brasiliensis*, *F. subtilis*, and *Litopenaeus schmitti* from the Brazilian coast were analyzed, and the obtained TC values in ethanol:acetone (1:1) shrimp extracts were between 2 and 9 μg/g [28]. Furthermore, a TC of 43 μg/g was obtained when using the shrimp species *Litopenaeus vannamei* from Brazil and the extraction performed in n-hexane:isopropanol (60:40) [29]. Another study presented similar TC values to the ones described in the present study: TC of 80 μg/g for *Trachysalambria curvirostris* [30]. This species was sampled on the coast of China, and the extraction solvent used was acetone. 

β-carotene values were higher in spring, and concerning the shrimp species, β-carotene and astaxanthin were higher in *P. serratus*. Astaxanthin mean values ranged between 1.1 and 26.1 μg/g dw in this study, and a value of 5.8 μg/g of astaxanthin in the shells of *Pandalus borealis* shrimp species, using ethanol and ultrasound equipment for the extraction, was reported [31]. The value reported is in the range of the values reported in the present study, and in the case of *P. serratus*, the values have higher printing value in their potential waste. High values of carotenoids, such as β-carotene and astaxanthin, have been linked to lower risks of cardiovascular and degenerative diseases [26], so the consumption or use of products with high values of these carotenoids could be beneficial to health.

The biological activity of the extracts varied between the analysis performed, ABTS^•+^ and ORAC values were higher in spring, whereas FRAP and DPPH^•^ values were higher in autumn. Concerning the shrimp species, DPPH^•^, ABTS^•+^, and ORAC values were higher in *P. varians*. That was the species from the estuary, so from brackish waters. Values of FRAP reported previously for shrimp shells were around 7.3 and 16.3 mg TE/g [32]. In that study [32], shrimps were from a Canadian company, and the solvent used was an ethylenediaminetetraacetic acid buffer. Regarding the shrimp muscle, the values were around 1.5 mg TE/g [33], and this result was obtained from the shrimp specie *Pandalus borealis* caught in the Norwegian sea. These results valorize the shrimp shell waste, and the values reported in the present study are similar to the values described for shrimp shells. Previous results for DPPH^•^ scavenging activity in shrimp shell waste were around 3 and 4.5 mg/g [32], slightly above the values shown in the present study. Regarding ABTS^•+^, values around 6 and 13 mg TE/g were reported [32], which are in accordance with the values described in the present work. Concerning ORAC for shrimp muscle samples, the value reported is around 38.8 mg TE/g [33]. This value is lower than the values reported in this study for shrimp shells, which valorizes shrimp shell waste.

Positive or negative correlations were found between some of the parameters analyzed. Positive correlations were found between TPC and FRAP values, and this was described previously in herbs, spices, and pulses [34,35]. This indicates that phenolic compounds could be major contributors to antioxidant power. In the same way, when TC values raise, astaxanthin values also increase in the shrimp shell samples, this could indicate that the total value of carotenoids is particularly influenced by the amount of the carotenoid astaxanthin. Negative correlations were found between DPPH^•^ values and astaxanthin. This was an unexpected result because astaxanthin is known for its antioxidant capacity. Nevertheless, a negative correlation between DPPH and other carotenoids was described previously for fruit samples [36]. Similarly, a negative correlation was found between FRAP and β-carotene. A previous study reported a negative correlation between FRAP and carotenoids [37]. In the case of the present work, the negative correlation was particularly with carotenoid β-carotene.

Regarding the study of shrimp shell waste as a potential enzyme inhibitor, a previous work studied a shrimp shell deproteinized bioactive hydrolysate from *Metapenaeus monoceros.* Using this hydrolysate, the authors reached 100% of AChE inhibition at 620 μg/mL and 100% of α-amylase inhibition at 400 μg/mL [38]. Conversely, in the present study, similar concentrations only inhibited 15% and 10% of AChE and α-amylase activity, respectively. Moreover, the acetone and dichloromethane extracts from by-products (heads, shells, and tails) of *Pandalus borealis*, *Pandalus hypsinotus*, and *Pandalopsis japonica*, characterized by carotenoid contents between 6.79 and 11.12 mg/g, displayed moderate AChE and BuChE inhibitions [39]. The authors reported IC_50_ in the range of 200–940 μg/mL against AChE and 670–2050 μg/mL against BuChE. These differences could be explained by the different extraction procedures used or can be justified by the differences between the shrimp species itself.

*P. varians* demonstrated good antimicrobial activity against *B. cereus*, *P. aeruginosa*, and *S. enteritidis*. Instead, *P. serratus* did not demonstrate any antimicrobial activity. These phenomena could be explained by the fact that *P. varians* is the specie from the Sado estuary. Brackish water, probably with high levels of bacteria, may increase the antimicrobial activity by adaptive responses, and although the shrimps have the same genera, they are classified into two different species and so can exhibit different antimicrobial activity [40]. Shrimp shell from a fish market in Dammam city, Saudi Arabia was extracted using methanol and previously evaluated [41] against some of the bacteria tested in the present study (*E. coli*, *S. aureus*, and *P. aeruginosa*), and the results for *P. aeruginosa* (18 mm) were similar to the presented results for *P. varians* from aquaculture origin. Concerning *S. aureus*, the results are similar to the previous results reporting no formed inhibition zone. However, the shrimp shell extract was not able to form an inhibition zone for *E. coli*, whereas the study from Gumgumjee et al. [41] presented a value of 30 mm, which can probably be explained by differences between the strains or extraction procedures [41].

## 5. Conclusions

The analysis of bioactive compounds from natural sources is nowadays of high importance. Shrimp shell waste analyzed in this study presented a significant antioxidant capacity with values in the range of previous works reported. The results demonstrate that, for the parameters evaluated, significant differences between the variables analyzed (species, seasons, and locations of sampling) were observed. All the findings of this work reveal the potential application of shrimp shell waste in new bioproducts. This incorporation could remarkably raise the antioxidant capacity of the products and improve their color making them more appealing to the food industry.

## Figures and Tables

**Table 1:** Total phenolic content (TPC) and total carotenoids (TC); (A)-aquaculture; (W)-wild; GAE-gallic acid equivalents.

Location of Sampling and Season	Species	TPC			TC		
		mg GAE/g			μg/g		
Figueira da Foz, Spring	*Palaemon* *serratus*	8.1	±	0.7	80	±	9
Figueira da Foz, Autumn		9	±	1	134	±	11
Vila do Conde, Spring		6.4	±	0.4	52	±	3
Vila do Conde, Autumn		10.4	±	0.7	127	±	8
Sado estuary (A), Spring	*Palaemon* *varians*	5.0	±	0.4	50	±	3
Sado estuary (A), Autumn		9.0	±	0.5	28	±	5
Sado estuary (W), Spring		4.7	±	0.3	42	±	2
Sado estuary (W), Autumn		9.9	±	0.6	32	±	3

**Table 2:** FRAP, RSA, and ORAC.; (A)-aquaculture; (W)-wild; AAE-ascorbic acid equivalents; TE-Trolox equivalents.

Location of Sampling and Season	Species	FRAP			DPPH				ABTS				ORAC		
		mg AAE/g			mg TE/g			IC_50_ (mg/mL)	mg TE/g			IC_50_ (mg/mL)	mg TE/g		
Figueira da Foz, Spring	*Palaemon* *serratus*	6	±	1	0.5	±	0.1	5.9	6	±	2	0.5	76	±	14
Figueira da Foz, Autumn		6	±	2	0.5	±	0.2	2.0	7	±	2	0.5	75	±	23
Vila do Conde, Spring		3	±	2	0.7	±	0.3	4.9	6	±	2	0.3	72	±	8
Vila do Conde, Autumn		7	±	1	0.5	±	0.1	4.3	4	±	2	0.6	94	±	35
Sado estuary (A), Spring	*Palaemon* *varians*	5	±	1	0.4	±	0.1	8.2	11	±	4	0.3	96	±	17
Sado estuary (A), Autumn		6	±	2	1.2	±	0.2	1.7	7	±	2	0.5	79	±	13
Sado estuary (W), Spring		5.1	±	0.7	0.7	±	0.2	3.6	6	±	4	0.3	130	±	21
Sado estuary (W), Autumn		6	±	2	0.9	±	0.3	2.0	7	±	2	0.5	87	±	20

## Data Availability

Not applicable.

## Supplementary Materials

The following supporting information can be downloaded at: https://www.mdpi.com/article/10.3390/antiox12020435/s1. Figure S1. Chromatogram of the sample Vila do Conde spring containing astaxanthin (1) and β-carotene (2) obtained in the PDA detector; Figure S2. Differences of β-carotene content between seasons, species, and locations; Figure S3. Differences of astaxanthin content between seasons, species, and locations; Figure S4. Differences of FRAP content between seasons, species, and locations; Figure S5. Differences of ORAC content between seasons, species, and locations; Table S1. Average diameter of inhibition zone (mm) of shrimp shell; Table S2. Positive and negative strong correlations between the elements analyzed.
