# Supplementary material for: Bioactive Compounds of Shrimp Shell Waste from Palaemon serratus and Palaemon varians from Portuguese Coast
Source: Antioxidants (Basel). 2023 Feb 9;12(2):435. doi: 10.3390/antiox12020435 (PMC9952640; doi:10.3390/antiox12020435)
Supplement: Supplementary file 1 [file antioxidants-12-00435-s001.zip › antioxidants-2164313-supplementary.pdf]

Supplementary Materials

Bioactive Compounds of Shrimp Shell Waste from *Palaemon serratus* and *Palaemon varians* from Portuguese Coast

Maria Luz Maia[1], Clara Grosso[1], Fátima Barroso[1], Aurora Silva[1,2], Cristina Delerue-Matos[1], Valentina Fernandes Domingues[1]

[1] REQUIMTE/LAQV, ISEP, Polytechnic of Porto, Dr. António Bernardino de Almeida, 4249-015 Porto, Portugal

[2] Nutrition and Bromatology Group, Department of Analytical and Food Chemistry, Faculty of Food Science and Technology, Ourense Campus, University of Vigo, E32004 Ourense, Spain

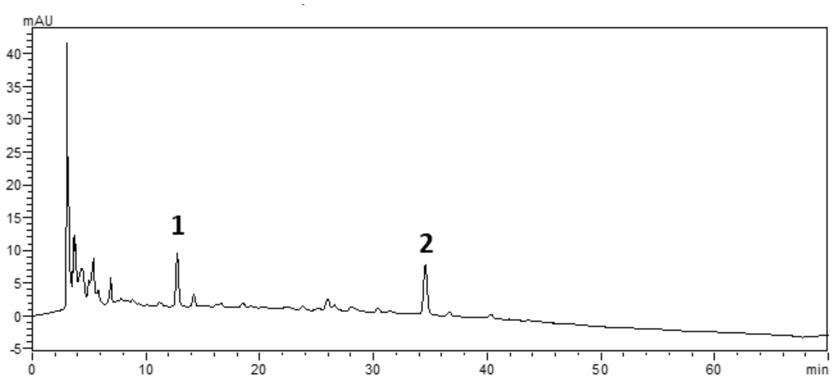

Figure S1. Chromatogram of the sample Vila do Conde spring containing astaxanthin (1) and β-carotene (2) obtained in the PDA detector.

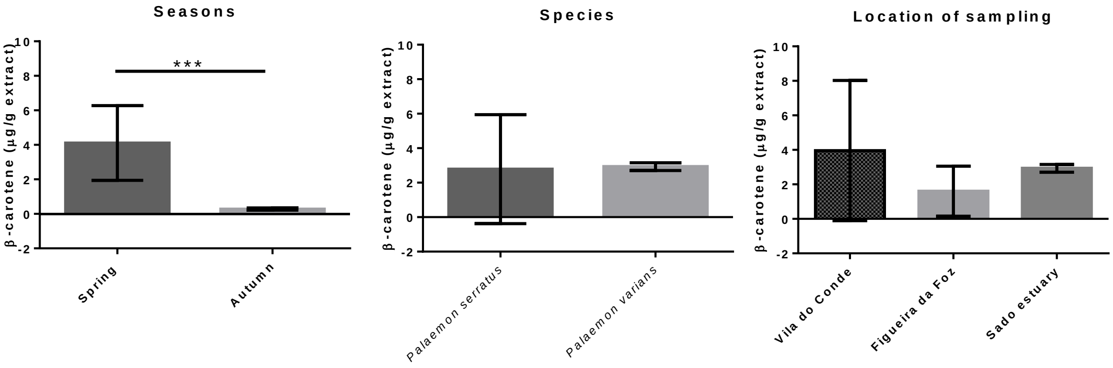

Figure S2. Differences of β-carotene content between seasons, species and locations. Columns correspond to mean of the concentration ± SD. Significantly different, ***p < 0.001.

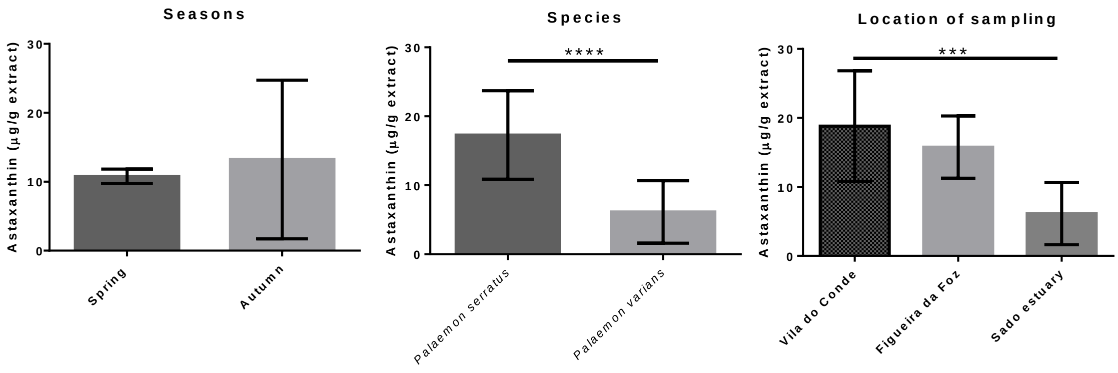

Figure S3. Differences of astaxanthin content between seasons, species and locations. Columns correspond to mean of the concentration ± SD. Significantly different, ****p < 0.0001, ***p < 0.001.

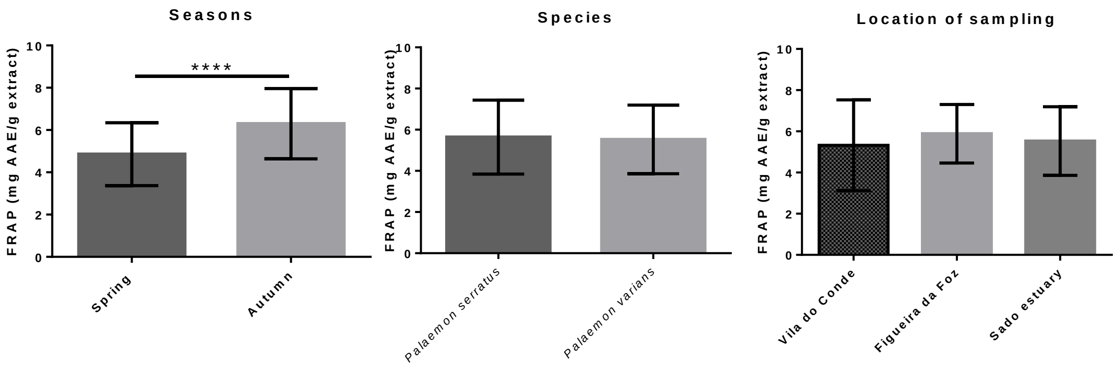

Figure S4. Differences of FRAP content between seasons, species and locations. Columns correspond to mean of the concentration ± SD. Significantly different, ****$p < 0.0001$.

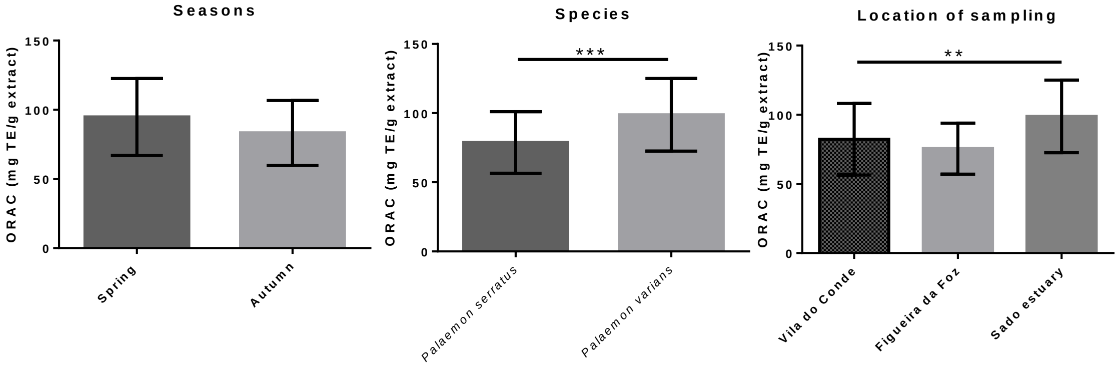

Figure S5. Differences of ORAC content between seasons, species and locations. Columns correspond to mean of the concentration ± SD. Significantly different, ***$p < 0.001$, **$p < 0.01$.

Table S1. Average diameter of inhibition zone (mm) of shrimp shell; (A)-aquaculture; (W)-wild.

Location of sampling and season	Species	*E. coli*	*S. aureus*	*B. cereus*	*P. aeruginosa*	*S. enteritidis*
**Figueira da Foz, Spring**	***Palaemon serratus***	-	-	-	-	-
**Figueira da Foz, Autumn**		-	-	-	-	-
**Vila do Conde, Spring**		-	-	-	-	-
**Vila do Conde, Autumn**		-	-	-	-	-
**Sado estuary (A), Spring**	***Palaemon varians***	-	-	21 ± 4	16 ± 2	20 ± 3
**Sado estuary (A), Autumn**		-	-	23.3 ± 0.3	20 ± 1	18 ± 13
**Sado estuary (W), Spring**		-	-	-	19.4 ± 0.4	-
**Sado estuary (W), Autumn**		-	-	-	14 ± 4	-

Table S2. Positive and negative strong correlations between the elements analyzed.

**Positive correlations**	**r**
TPC with FRAP	0.78
TC with astaxanthin	0.92
**Negative correlations**	
DPPH with astaxanthin	0.71
FRAP with β-carotene	0.91
